# NAIAD-2020: Characteristics of Motor Evoked Potentials After 3-Day Exposure to Dry Immersion in Women

**DOI:** 10.3389/fnhum.2021.753259

**Published:** 2021-12-01

**Authors:** Inna Nosikova, Alexandra Riabova, Liubov Amirova, Vladimir Kitov, Elena Tomilovskaya

**Affiliations:** Laboratory of Gravitational Physiology of the Sensorimotor System, Institute of Biomedical Problems of Russian Academy of Science, Moscow, Russia

**Keywords:** Dry Immersion, NAIAD-2020, microgravity, TMS, support unloading, trans-spinal magnetic stimulation

## Abstract

As female astronauts participate in space flight more and more frequently, there is a demand for research on how the female body adapts to the microgravity environment. In particular, there is very little research on how the neuromuscular system reacts to gravitational unloading in women. We aimed to estimate changes in motor evoked potentials (MEPs) in the lower leg muscles in women after 3-day exposure to Dry Immersion (DI), which is one of the most widely used ground models of microgravity. Six healthy female volunteers (mean age 30.17 ± 5.5 years) with a natural menstrual cycle participated in this experiment. MEPs were recorded from the gastrocnemius and soleus muscles twice before DI, on the day of DI completion, and 3 days after DI, during the recovery period. To evoke motor responses, transcranial and trans-spinal magnetic stimulation was applied. We showed that changes in MEP characteristics after DI exposure were different depending on the stimulation site, but were similar for both muscles. For trans-spinal stimulation, MEP thresholds decreased compared to baseline values, and amplitudes, on the contrary, increased, resembling the phenomenon of hypogravitational hyperreflexia. This finding is in line with data observed in other experiments on both male and female participants. MEPs to transcranial stimulation had an opposing dynamic, which may have resulted from the small group size and large inter-subject variability, or from hormonal fluctuations during the menstrual cycle. Central motor conduction time remained unchanged, suggesting that pyramidal tract conductibility was not affected by DI exposure. More research is needed to explore the underlying mechanisms.

## Introduction

Nowadays women participate in space flights (SF) alongside men, and the differences between the sexes should be considered when training crewmembers for space missions. For many years space physiology studies have been conducted with male volunteers (Kozlovskaya and Kirenskaya, 2004; Koppelmans et al., 2015, 2017; De Abreu et al., 2017; Amirova et al., 2020), which was the “gold standard” of research, and was reasonable at the dawn of manned space exploration. However, physiological changes triggered in the female body by microgravity have been insufficiently investigated, and this may lead to both employment and casual discrimination of female astronauts.

One of the important lines of research in space physiology is studying how weightlessness affects motor system function. Preventing and predicting motor impairments caused by weightlessness is especially significant for increasing SF duration and expanding the scope of motor tasks performed during space missions. The complex of changes occurring in human motor function under the conditions of real or simulated microgravity is called hypogravitational motor syndrome (Kozlovskaya et al., 1988), and it is defined by a deficit in vestibular, proprioception, and support afferent activity (Pechenkova et al., 2019), and substantial alterations in the functional (e.g., atony, a decline in speed-force qualities) and structural (e.g., atrophy and a phenotype deterioration) characteristics of skeletal muscles (Kozlovskaya et al., 1988; Kozlovskaya and Kirenskaya, 2004; Koppelmans et al., 2017; Amirova et al., 2020). In experiments with animal models it was also shown that a hindlimb suspension in rats results in nerve fiber demyelinization, which in turn may play a role in the development of hypogravitational motor syndrome (Islamov et al., 2013).

Because invasive techniques to study the brain and the spinal cord under the conditions of support withdrawal cannot be used, a different method is required. Recently transcranial magnetic stimulation (TMS) began to be utilized in the field of space medicine and biology (Davey et al., 2004; Roberts et al., 2007, 2010; Badran et al., 2020; Romanella et al., 2020; Nosikova et al., 2021). This method is widely used in studying cognition, brain-behavior relationships and the pathophysiology of neurological and psychiatric disorders; in particular, TMS of the motor cortex has a well-established role in clinical neurophysiology. Stimulation is achieved by applying electromagnetic induction to generate suprathreshold current in the brain, and different shaped TMS coils allow stimulation of both deep structures and selected small regions of the cortex. TMS variables that are typically analyzed in clinical and research studies include motor thresholds, motor evoked potential (MEP) amplitudes, and MEP latencies among others. Motor threshold is the minimal intensity of stimulation required to elicit a reliable MEP of minimal amplitude in the target muscle. Thresholds are measured to estimate cortical and spinal neurons excitability and they depend on a number of factors such as coil position and orientation, the individual arousal level, and environmental noise. MEP amplitudes, which reflect muscle contraction magnitude to a select stimulation intensity, are also widely used to study corticospinal excitability. Lastly, MEP latency is the time interval from the stimulus onset to the muscle response. The difference between latencies to stimulation of the motor cortex and spinal roots, called central motor conduction time (CMCT), is calculated to estimate corticospinal conductibility (Rossini et al., 2015). It was reported that MEPs to TMS depend on physical individual features. Specifically, MEP latency increases with age and positively correlates with height. Moreover, females show smaller latencies in upper limbs to both cortical and spinal stimulation when compared to males (Cantone et al., 2019).

Earlier studies were more focused on the female cardiovascular system’s reaction to microgravity (Demiot et al., 2007; Hodges et al., 2010; Arbeille et al., 2012; Edgell et al., 2012), and it was shown that female astronauts are more susceptible to orthostatic intolerance after SF than male astronauts (Platts et al., 2014). A few research papers report postural performance impairments (Viguier et al., 2009) and muscle atrophy (Shenkman et al., 2000) after head-down bed rest in women, but the authors do not compare their results to male groups. It was also reported that women are more likely to suffer from space motion sickness and vestibular instability after SF than men (Reschke et al., 2014). As for neuromuscular changes, long-term gravitational unloading leads to less deterioration of muscle force characteristics in females compared to males, but on the other hand, males are better at integrating different sensory inputs when performing explosive motor tasks after unloading (Koryak, 2009). However, there is still very little research on the state of the female neuromuscular system after exposure to real SF or ground-based models, which makes it difficult to select more effective countermeasures for female groups (Holt et al., 2016).

We hypothesized that, as there appear to be some differences in neuromuscular adaptation to support withdrawal between men and women, the motor responses evoked by magnetic stimulation (MS) will possess different characteristics after exposure to simulated microgravity in women compared to men. Thus, we decided on Dry Immersion (DI) as one of the most widely used ground models of microgravity and carried out a 3-day experiment on a group of female volunteers to estimate how their MEP characteristics changed.

## Materials and Methods

### Participants

Six healthy female volunteers (mean age 30.17 ± 5.5 years) of reproductive age participated in this study. The participants had similar bodily constitution, height (166.6 ± 3.3 cm) and body weight (62.0 ± 3.4 kg). All subjects had a natural menstrual cycle and no history of motor impairments or neurological diseases. Each participant signed an informed consent after the experimental procedures and possible consequential effects and risks were explained to them.

The present study was approved by the Bioethical Commission of the Institute of Biomedical Problems of Russian Academy of Science (Protocol No. 544 of July 16, 2020) and fully complied with the principles of the Declaration of Helsinki.

### Experimental Design

The study was conducted at the DI facility of the Institute of Biomedical Problems, Russian Academy of Science (Tomilovskaya et al., 2019, 2021). For 3 days, participants lay in the immersion bath without any physical activities and with moderate movement restriction; among other factors, lower limb activation was limited. The water temperature in the bath was maintained at 32.5 ± 2°C. Every evening the subjects were lifted out of the bath for 15–20 min for hygiene procedures, the majority of which were performed in the supine position. The subjects were also raised from the bath during the day for certain experimental examinations that were carried out in the supine position. The average time spent outside the immersion bath did not exceeded 30 min per day. The crew, consisting of a doctor, an assistant and a technician, provided 24-h monitoring of the participants’ health and the working condition of technical equipment. In their free time, subjects were allowed to read, work on a laptop, watch TV, talk on the phone, etc.

### Magnetic Stimulation Procedure

**FIGURE 1:**
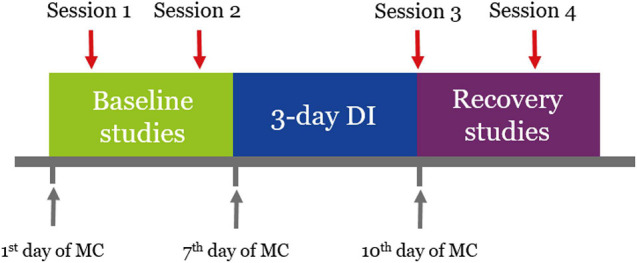
Schedule of the experiment. Sessions 1 and 2 were conducted 5 and 3 days before the start of DI. Session 3 was conducted on the day of DI completion. Session 4 was conducted 3 days after DI. Every experimental stage started on a specific day of the MC.

Experimental sessions were conducted according to the schedule (Figure 1). There were four sessions in total: two before DI (5 and 3 days before the start of DI, baseline studies), one right after DI (on the day of DI completion, referred to as R + 0), and one during the recovery period (on the third day after DI, referred to as R + 3). The start of every experimental stage was matched with a specific day of the menstrual cycle (MC).

Participants were instructed to abstain from alcoholic and tonic drinks the day before each procedure. During the procedure, the subjects lay prone, relaxed and with their eyes open. A support was placed under the ankles for better relaxation. Motor responses were obtained using transcranial and trans-spinal MS, and MEPs were recorded from the soleus and gastrocnemius muscles of the right leg.

Transcranial MS was delivered with the 8-shaped coil (DB-80 Butterfly) of the MagPro X100 magnetic stimulator (Medtronic, Denmark) to the area of cortical motor projections of the right lower leg muscles. The coil was placed 1–2 cm to the left from the intersection of the vertex and the line connecting the pre-auricular points and then was gradually moved to the position at which stimulation led to MEPs with the greatest amplitude and a constant shape. Trans-spinal MS was delivered using a flat round coil with an outer diameter of 114 mm, which was placed at the level of L5–S1 segments of the lumbar spine. If the stimulation area was picked correctly, MEP amplitudes were generally stable, which means MEPs had a constant shape and their amplitudes were similar. Motor responses of soleus and gastrocnemius muscles were recorded with bipolar surface silver-chloride electrodes that were placed in the center of the muscle belly projections with a 20 mm interelectrode distance. Electromyographic signals were recorded using a Viking Quest 4-channel myograph (Viasys, United States) with a 2 Hz to 10 kHz passband. The sensitivity band was 0.1 μV to 10 mV; the input noise did not exceeded 40 μV.

After obtaining the coil positioning which ensured stable motor responses, we first retrieved MEP thresholds by decreasing the stimulation magnitude in steps of 2–5% of maximal output and stimulating the target area with an interval of more than 3 s. The magnitude that evoked responses of 20–50 μV amplitude with a 50 or more percent probability was taken as a threshold (Nikitin and Kurenkov, 2003). The muscle relaxation during thresholds evaluation was monitored *via* real-time electromyogram (EMG). We then increased stimulation magnitude in steps of 5–10% of maximal output until reaching maximal MEP amplitudes or 100% of maximal output. At each step, we recorded at least three MEPs. MEPs to transcranial and trans-spinal MS are referred to as “cortical MEPs” and “spinal MEPs,” respectively.

### Data Processing and Statistical Analysis

Motor evoked potential data were extracted from muscle curves using Viking Quest 11.1 software, raw latency and amplitude values for each single stimulation were obtained. For each participant we evaluated MEP thresholds and mean maximal peak-to-peak amplitudes at three registration points: baseline (average of two baseline points), R + 0, and R + 3.

For demonstration purposes, threshold and amplitude values were presented as mean ± SEM of percent changes from baseline, which was taken as zero. Statistical analysis was performed with GraphPad Prism 8 software. Data normality was assessed using the Kolmogorov–Smirnov test. Because data were generally not normally distributed, threshold and amplitude mean values were compared using the Friedman test with *post hoc* Dunn’s multiple comparisons test. Data were assumed statistically significant at *p* < 0.05.

We also evaluated CMCT, which is calculated with the following formula: CMCT = cortical MEP latency − spinal MEP latency. Latency was measured as the time interval between the MS artifact and the first deflection of the muscular response from EMG baseline. From a series of responses with maximal amplitudes, the MEP with the shortest latency was considered for CMCT calculation.

## Results

Both thresholds and amplitudes of MEPs showed differences in range and direction of changes between subjects after DI and during the recovery period (Supplementary Table 1). Due to this inter-subject variance, value shifts on R + 0 and R + 3 were mostly not statistically significant compared with baseline. Nevertheless, there were general tendencies present in the majority of the group.

**FIGURE 2:**
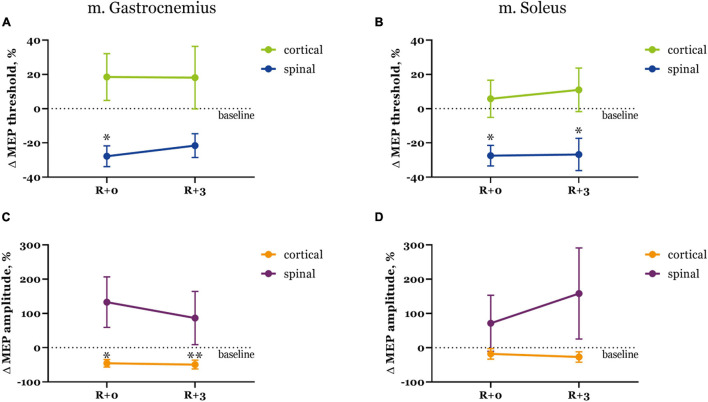
Motor evoked potential thresholds **(A,B)** and MEP amplitudes **(C,D)** in the gastrocnemius **(A,C)** and soleus **(B,D)** muscles. Data is presented as mean ± SEM of percent changes from baseline. Baseline values are taken as zero. R + 0 – day of DI completion, R + 3 – third day after DI. ^∗^*p* < 0.05 vs. baseline, ^∗∗^*p* < 0.01 vs. baseline.

Threshold values changed in a similar manner for both recorded muscles, but depended on the stimulation site (Figures 2A,B). Specifically, spinal MEP thresholds lowered right after DI and then exhibited a slight recovery, more so in the gastrocnemius muscle. Cortical MEP thresholds conversely were higher right after DI and in the soleus muscle they even showed a tendency to increase during the recovery period. The decrease in thresholds to trans-spinal MS was more prominent (−27.7 and −27.5% from baseline in the gastrocnemius and soleus muscles respectively at R + 0) than the increase in thresholds to transcranial MS (18.5 and 5.8% from baseline in the gastrocnemius and soleus muscles, respectively at R + 0). Only spinal MEP thresholds significantly reduced from baseline (*p* < 0.05) after DI.

Interestingly, changes in MEP amplitudes were opposite for both types of stimulation (Figures 2C,D). Cortical MEPs had smaller amplitudes right after DI compared with baseline (−45.4 and −18.0% decrease in the gastrocnemius and soleus muscles, respectively), and their amplitudes decreased even further by the third day of recovery (−49.4 and −27.0% from baseline in gastrocnemius and soleus muscles, respectively). Spinal MEPs were characterized by substantially larger amplitudes on R + 0 (132.8 and 71.6% increase from baseline in gastrocnemius and soleus muscles, respectively) which then decreased to 86.5% in the gastrocnemius muscle and increased to 158.3% in the soleus muscle during the recovery period. Again, as was described for the threshold values, changes in MEP amplitudes were more drastic for trans-spinal MS.

**FIGURE 3:**
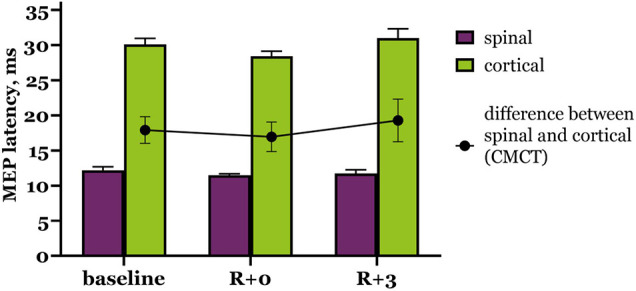
Motor evoked potential latency to trans-spinal (purple bars) and transcranial (green bars) MS. Black dots represent CMCT values. R + 0 – day of DI completion, R + 3 – third day after DI.

We also calculated CMCT (Supplementary Table 2) to estimate possible changes in pyramidal tract conductibility. As shown on Figure 3, CMCT did not change significantly under the condition of DI, although it slightly decreased right after DI and then increased beyond baseline during the recovery period. Cortical MEP latencies had the biggest contribution to these CMCT fluctuations.

## Discussion

Evoked responses generally changed in the same direction after DI regardless of the muscle being mostly phasic (gastrocnemius) or tonic (soleus). On the other hand, there were completely different dynamics of changes dependent on the stimulation site, i.e., cranial or spinal placement of the coil.

Motor evoked potential characteristics for trans-spinal MS after DI exposure (i.e., decreased thresholds and increased amplitudes) resemble the phenomenon of hypogravitational hyperreflexia – muscle hyperreflexia developing in the microgravity environment (Kozlovskaya et al., 1988). Previously we carried out a similar experiment on a group of male subjects (Nosikova et al., 2021) and described the same spinal MEPs changes. An increase in spinal excitability has also been shown in unilateral lower limb suspension, the other ground-based model of microgravity. The experiments were carried out on mixed groups, included men and women, and after 4 weeks of unloading an increase in the soleus H-reflex was observed (Clark et al., 2006, 2007). This evidence shows that hypogravitational hyperreflexia probably has a spinal origin and that it develops regardless of the subject’s sex.

Cortical MEPs in women had the opposite dynamic: for most subjects their thresholds increased and amplitudes became smaller after DI. However, in men cortical MEPs changed with the same dynamic as spinal MEPs throughout the experiment, although shifts to transcranial MS were not as severe (Nosikova et al., 2021). TMS studies by other authors in this field are scarce, and their results appear to be inconsistent. For instance, in a parabolic flight induced weightlessness a facilitation of MEP responses was reported, suggesting an increase in corticospinal excitability during 0 G (Davey et al., 2004). In a similar study on a small male group zero gravity also led to a decrease in MEP thresholds, and the authors provided a few possible explanations for the observed phenomenon, including corticospinal excitability increase (Badran et al., 2020). Such changes in excitability were also observed after 10 days of lower leg immobilization (Roberts et al., 2007), although the authors hypothesize that excitability increases because of motor recovery and re-learning. Additionally, there were no significant differences in resting motor thresholds across experimental sessions. By contrast, in a long-term bed rest study corticospinal excitability decreased in the immediate post-bed rest period (Roberts et al., 2010). It is important to note that both studies by Roberts et al. (2010) were conducted on mixed groups, but no comparison between male and female participants was made.

Considering the literature, it is hard to tell whether the difference between male and female groups in two our experiments was sex dependent. The general decrease in amplitudes and increase in thresholds of cortical MEPs in women suggests a decrease in corticospinal excitability, but since such changes were not present in men (Nosikova et al., 2021), we cannot confidently conclude that corticospinal excitability reduces as a function of DI or support withdrawal. There is also an inconsistency in the dynamics of MEP characteristics in different models of microgravity (Davey et al., 2004; Roberts et al., 2007, 2010; Badran et al., 2020). It is possible that the small group size and large inter-subject variability affected our results.

We can also take into account hormonal fluctuations during the MC, which may affect nervous control of muscle activity. For example, estrogen has an excitatory impact on the nervous system, and progesterone induces inhibition, resulting in shifts of neuromuscular function throughout the MC (Ansdell et al., 2019). We suppose that changes in the endocrine profile do not greatly affect spinal conductibility, as spinal MEPs appear to be similar in men and women, although MEP amplitudes in women varied substantially across subjects (Figures 2C,D). Spinal MEP latencies also were unchanged after DI (Figure 3), which proves that spinal conductibility was not affected. Cortical MEPs pose a more challenging question about the origin of their changes that could be influenced by the MC. Specifically, varying dynamics of MEP characteristics between subjects could signify diverging adaptive reactions of neurons, therefore, one should judge the described changes carefully. More research is needed to explore and better understand the underlying mechanisms.

### Limitations

The main limitation of the study is the sample size of 6 as it is too low to provide reliable outcomes measures. However, this is a novel study, and we believe that the data obtained in the first female DI experiment might be helpful for the future research in this field.

## Conclusion

The results of our study show that 3-day support withdrawal in women leads to an increase in spinal excitability, which manifests as a threshold decline and amplitude increase of trans-spinal MS evoked motor responses in the lower leg muscles. These data are in line with our previous research conducted on a male group, as well as with studies carried out with the participation of both men and women. The changes in corticospinal excitability were ambiguous and could possibly be affected by a number of factors such as large inter-subject variability or the sex hormones profile. As this finding is not fully supported by the literature, it demands further and more careful research.

## Data Availability Statement

The raw data supporting the conclusions of this article will be made available by the authors, without undue reservation.

## Ethics Statement

The studies involving human participants were reviewed and approved by the Bioethical Commission of the Institute of Biomedical Problems of Russian Academy of Science. The patients/participants provided their written informed consent to participate in this study.

## Author Contributions

IN and AR collected the data and wrote the draft of the manuscript. LA contributed to the data analysis and design of the figures. VK contributed with the technical support. ET made a revision of the manuscript and was a supervisor of the study. All authors contributed to the article and approved the submitted version.

## Conflict of Interest

The authors declare that the research was conducted in the absence of any commercial or financial relationships that could be construed as a potential conflict of interest.

## Publisher’s Note

All claims expressed in this article are solely those of the authors and do not necessarily represent those of their affiliated organizations, or those of the publisher, the editors and the reviewers. Any product that may be evaluated in this article, or claim that may be made by its manufacturer, is not guaranteed or endorsed by the publisher.
