# Supplementary material for: NAIAD-2020: Characteristics of Motor Evoked Potentials After 3-Day Exposure to Dry Immersion in Women
Source: Front Hum Neurosci. 2021 Dec 1;15:753259. doi: 10.3389/fnhum.2021.753259 (PMC8671694; doi:10.3389/fnhum.2021.753259)
Supplement: Supplementary file 1 [file Table_1.DOCX]

Supplementary Table 1. Raw and baseline-corrected values of MEP thresholds and MEP maximal amplitudes to transcranial and trans-spinal MS.

|  | Transcranial MS | | | | | | Trans-spinal MS | | | | | |
| --- | --- | --- | --- | --- | --- | --- | --- | --- | --- | --- | --- | --- |
|  | MEP threshold, % | | | MEP max amplitude, µV | | | MEP threshold, % | | | MEP max amplitude, µV | | |
|  | baseline | R+0 | R+3 | baseline | R+0 | R+3 | baseline | R+0 | R+3 | baseline | R+0 | R+3 |
| m. Gastrocnemius | | | | | | | | | | | | |
| Raw data  Participants 1-6 | 75 | 85 | 85 | 48.75 | 47.75 | 36 | 45 | 25 | 30 | 3312.75 | 9708.75 | 3079.50 |
|  | 55 | 70 | 48 | 372.87 | 232.50 | 167 | 85 | 75 | 65 | 90.50 | 225 | 92.50 |
|  | 67 | 65 | 67 | 283.75 | 143 | 187 | 50 | 43 | 50 | 254.25 | 318.50 | 334.75 |
|  | 100 | 85 | 100 | 145.75 | 37 | 20.25 | 65 | 45 | 55 | 93.75 | 104.50 | 107.50 |
|  | 60 | 65 | 60 | 104.25 | 66 | 93.25 | 50 | 40 | 45 | 232.75 | 148.25 | 242 |
|  | 25 | 45 | 52 | 1340 | 375.50 | 209.75 | 55 | 30 | 29 | 234 | 1298.50 | 1341.50 |
| Baseline-corrected data  Participants 1-6 | 0 | 13.33 | 13.33 | 0 | -2.05 | -26.15 | 0 | -44.44 | -33.33 | 0 | 193.07 | -7.04 |
|  | 0 | 27.27 | -12.73 | 0 | -37.65 | -55.21 | 0 | -11.76 | -23.53 | 0 | 148.62 | 2.21 |
|  | 0 | -2.98 | 0 | 0 | -49.60 | -34.10 | 0 | -14 | 0 | 0 | 25.27 | 31.67 |
|  | 0 | -15 | 0 | 0 | -74.61 | -86.11 | 0 | -30.77 | -15.38 | 0 | 11.47 | 14.67 |
|  | 0 | 8.33 | 0 | 0 | -36.69 | -10.55 | 0 | -20 | -10 | 0 | -36.30 | 3.97 |
|  | 0 | 80 | 108 | 0 | -71.98 | -84.35 | 0 | -45.45 | -47.27 | 0 | 454.91 | 473.29 |
| mean ± SEM |  | 18.49 ± 13.63 | 18.10 ± 18.29 |  | -45.43 ± 10.94 | -49.41 ± 12.76 |  | -27.73 ± 6.07 | -21.58 ± 6.92 |  | 132.84 ± 73.60 | 86.46 ± 77.55 |
| m. Soleus | | | | | | | | | | | | |
| Raw data  Participants 1-6 | 95 | 85 | 100 | 51.50 | 63 | 65.50 | 45 | 25 | 19 | 3664 | 4818.50 | 1962.50 |
|  | 55 | 70 | 48 | 242.75 | 316.75 | 240.25 | 90 | 80 | 60 | 112.50 | 116.75 | 335.50 |
|  | 67 | 60 | 67 | 271.25 | 226.50 | 232 | 50 | 43 | 50 | 412.75 | 336.87 | 385.50 |
|  | 100 | 85 | 100 | 112.75 | 59.25 | 37.25 | 65 | 50 | 55 | 263.50 | 160.50 | 317 |
|  | 60 | 56 | 60 | 326 | 125 | 119.50 | 60 | 44 | 56 | 194 | 145.25 | 167 |
|  | 30 | 45 | 52 | 516.25 | 336 | 293.25 | 55 | 30 | 29 | 233.25 | 1345.50 | 2095 |
| Baseline-corrected data  Participants 1-6 | 0 | -10.53 | 5.26 | 0 | 22.33 | 27.18 | 0 | -44.44 | -57.78 | 0 | 31.51 | -46.44 |
|  | 0 | 27.27 | -12.73 | 0 | 30.48 | -1.03 | 0 | -11.11 | -33.33 | 0 | 3.78 | 198.22 |
|  | 0 | -10.45 | 0 | 0 | -16.50 | -14.47 | 0 | -14 | 0 | 0 | -18.38 | -6.60 |
|  | 0 | -15 | 0 | 0 | -47.45 | -66.97 | 0 | -23.08 | -15.38 | 0 | -39.09 | 20.30 |
|  | 0 | -6.67 | 0 | 0 | -61.66 | -63.34 | 0 | -26.67 | -6.67 | 0 | -25.13 | -13.92 |
|  | 0 | 50 | 73.33 | 0 | -34.91 | -43.20 | 0 | -45.45 | -47.27 | 0 | 476.85 | 798.18 |
| mean ± SEM |  | 5.77 ± 10.85 | 10.98 ± 12.71 |  | -17.95 ± 15.32 | -26.97 ± 15.22 |  | -27.46 ± 6.00 | -26.73 ± 9.44 |  | 71.59 ± 81.68 | 158.29 ± 132.77 |
