# Supplementary material for: NAIAD-2020: Characteristics of Motor Evoked Potentials After 3-Day Exposure to Dry Immersion in Women
Source: Front Hum Neurosci. 2021 Dec 1;15:753259. doi: 10.3389/fnhum.2021.753259 (PMC8671694; doi:10.3389/fnhum.2021.753259)
Supplement: Supplementary file 2 [file Table_2.DOCX]

Supplementary Table 2. Mean MEP latency values and calculated CMCT for each participant.

|  | Cortical MEP latency, ms | | | Spinal MEP latency, ms | | | CMCT, ms | | |
| --- | --- | --- | --- | --- | --- | --- | --- | --- | --- |
|  | baseline | R+0 | R+3 | baseline | R+0 | R+3 | baseline | R+0 | R+3 |
| Participants 1-6 | 31 | 27.9 | 36.6 | 11.2 | 11.3 | 11.6 | 19.8 | 16.6 | 25 |
|  | 29.6 | 29.2 | 30.8 | 11.8 | 11.1 | 11.3 | 17.8 | 18.1 | 19.5 |
|  | 29.7 | 28.3 | 28.8 | 12.2 | 11.2 | 11.6 | 17.5 | 17.1 | 17.2 |
|  | 28.3 | 26.7 | 29 | 13.4 | 12.2 | 12.5 | 14.9 | 14.5 | 16.5 |
|  | 28.1 | 27 | 27.9 | 10.7 | 11.9 | 9.7 | 17.4 | 15.1 | 18.2 |
|  | 33.9 | 31.5 | 32.9 | 13.8 | 11.2 | 13.6 | 20.1 | 20.3 | 19.3 |
| mean ± SEM | 30.10 ± 0.87 | 28.43 ± 0.71 | 31.00 ± 1.33 | 12.18 ± 0.50 | 11.48 ± 0.18 | 11.72 ± 0.53 | 17.92 ± 0.77 | 16.95 ± 0.86 | 19.28 ± 1.24 |
